# Identification of Gαi3 as a novel molecular therapeutic target of cervical cancer

**DOI:** 10.7150/ijbs.77126

**Published:** 2022-09-06

**Authors:** Jie Zhang, De-pei Yin, Yan Zhang, Jia-nan Zhang, Yan Yang, Zhi-qing Zhang, Li Zhou, Yan Lv, Hai-wei Huang, Cong Cao

**Affiliations:** 1 Obstetrics and Gynecology Department, The Affiliated Zhangjiagang Hospital of Soochow University, Institute of Neuroscience, Soochow University, Suzhou, China.; 2 Department of Otorhinolaryngology Head and Neck Surgery, Children's Hospital of Soochow University, Suzhou, China.; 3 Department of Radiotherapy and Oncology, Affiliated Kunshan Hospital of Jiangsu University, Suzhou, China.; 4 Center of Translational Medicine, The Affiliated Zhangjiagang Hospital of Soochow University, Suzhou, China.

**Keywords:** Cervical cancer, Targeted therapy, Gαi3, Akt-mTOR

## Abstract

Here we studied expression and potential functions of Gαi3 in cervical cancer. The bioinformatics analysis together with the results from local patients' tissues revealed that Gαi3 expression was remarkably elevated in human cervical cancer tissues and different cervical cancer cells, and was associated with poor overall survival and poor disease-specific survival of patients. Gαi3 depletion resulted in profound anti-cervical cancer activity. In primary or immortalized cervical cancer cells, Gαi3 shRNA or CRISPR/Cas9-caused Gαi3 knockout/KO largely hindered cell proliferation and migration, and provoked apoptosis. On the contrast, ectopic Gαi3 overexpression further enhanced cervical cancer proliferation and migration. Akt-mTOR activation in primary cervical cancer cells was significantly reduced after Gαi3 silencing or KO, but was augmented following Gαi3 overexpression. Further studies revealed that the transcription factor GATA4 binding to Gαi3 promoter region was significantly enhanced in cervical cancer tissues and cells. Gαi3 expression was decreased by GATA4 shRNA, but upregulated following GATA4 overexpression. *In vivo*, the growth of cervical cancer xenografts was robustly suppressed after Gαi3 silencing or KO. Gαi3 depletion and Akt-mTOR inactivation were detected in Gαi3-silenced/-KO cervical cancer xenograft tissues. Together, upregulated Gαi3 is a valuable oncotarget of cervical cancer.

## Introduction

Cervical cancer seriously threatens women's health globally 1, 2. The number of cases in developing countries accounts for over 85% of the world. Although the screening (mainly HPV screening) of cervical cancer has been relatively complete 1, 2, and the surgical techniques, radiotherapy equipment, and chemotherapy have been gradually improved, the clinical treatment of advanced and recurrent cervical cancer is still unsatisfactory, and the prognosis is still poor 3-5. The chemotherapy response for advanced cervical cancer is between 20% and 36%, and the survival time is less than one year 3-5. Although significant achievements have been made in cervical cancer therapy, the overall survival/prognosis for recurrent and metastatic patients is extremely poor 3, 4.

Molecularly-targeted agents, including the anti-angiogenic drugs, immuno-suppressants and EGFR blockers, are being tested for advanced cervical cancer 6, 7. Bevacizumab combined with chemotherapy were shown to improve overall survival in certain advanced cervical cancer patients 6, 8-10. However, for many advanced cancers, the novel targeted therapies are still in urgent need 11-14.

Gαi proteins contain three primary subunits, Gαi1/2/3 15. Studies from our group have shown that Gαi proteins are essential novel proteins in transducing signals by receptor tyrosine kinases (RTKs) 16-22 and non-RTK receptors 17, 23. Gαi proteins mediate activation oncogenic signalings (PI3K-Akt-mTOR and Erk-MAPK) by associating with ligand-activated receptors (RTKs and others) 16-22. We have recently identified that Gαi proteins are elevated in different human cancers, essential for tumorigenesis and cancer progression 16, 20, 24-26. Gαi3's expression and potential functions cervical cancer are explored here.

## Materials and methods

### Reagents

The antibodies were reported early 20. LY294002 together other chemicals/reagents were provided by Sigma (St. Louis, Mo).

### Cells

The fresh cervical cancer tissues or the paracancerous epithelial tissues were first digested. The digested human cells were washed, centrifuged, and incubated in complete medium with penicillin/streptomycin and DNase (500 U). Cell suspensions were thereafter filtered, centrifuged, and resuspended. The primary cancer cells or cervical epithelial cells were cultivated in described medium 27 with minor modifications. Here, the primary human cervical cancer cells (“priCC-1” and “priCC-2”) and the primary human cervical epithelial cells (“priCEpi-1” and “priCEpi-2”), derived from same two primary patients, were obtained. The immortalized cervical cancer cell lines, Caski and HeLa229, were provided by the Cell Bank of Institute of Biological Science of CAS (Shanghai, China). The protocols of this study were approved from the Ethics Committee of Soochow University and were according to the principles of Helsinki declaration.

### Human tissues

The human tissues, including cervical cancer tissues and matched paracancerous normal cervical tissues, were obtained from a total of twenty patients who were administrated at the Affiliated Hospitals of Soochow University. Each single patient provided the written-informed consent. The patients' information was listed in **Table 1**. All cancers are squamous cell carcinomas. Tissue slides were subject to immunohistochemistry (IHC) staining (using the described protocols 16).

### Genetic modification of Gαi3

Gαi3 shRNA, Gαi3 knockout (KO) by using the established CRISPR/Cas9 strategy, as well as ectopic Gαi3 overexpression using a lentiviral construct were reported previously 16, 26.

### Genetic modification of GATA4

The lentiviral constructs encoding the GATA4 shRNA or the GATA4-expressing sequence, as well as their relative control constructs, were reported in our previous study 28. The constructs were individually and stably transduced to the primary human cervical cancer cells. Expression of GATA4 was always tested.

### Constitutively-active mutant Akt1* (caAkt1)*

In brief, the caAkt1 (S473D)-expressing lentivirus (see our previous studies 29, 30) was added to cultured primary human cervical cancer cells. caAkt1-expressing stable cells were then formed by using puromycin.

### Other assays

Cellular function assays, including CCK-8 (testing cell viability), nuclear EdU/DAPI staining (testing cell proliferation) and “Transwell” migration, as well as the nuclear TUNEL/DAPI staining, Annexin V-PI flow cytometry, Caspase-3 activity assay were reported early 16, 18, 26, 31. Gene and protein detections by quantitative real-time PCR (qRT-PCR) and Western blotting were reported early 19, 22, 26. The detailed protocols of GATA4 chromosome immunoprecipitation (ChIP) were reported previously 28. mRNA primers were reported previously 16, 26. The uncropped blotting images were listed in Figure **S1**.

### Animal studies

The nude mice were reported previously 24. priCC-1 cells, at seven million cells of each xenograft, were subcutaneously (*s.c.*) injected mice's flanks. Within three weeks cervical cancer xenografts were formed (~ 80 mm^3^). The intratumoral injection of Gαi3 shRNA AAV (adeno-associated viruses) or scramble control shRNA AAV was reported previously 26. The IHC staining of xenograft slides were reported early 16, 20. The measurement of tumors was reported early 26. Soochow University's Ethics Committee and IACUC reviewed the protocols.

### Statistical analyses

*In vitro* experiments here were repeated five times with similar results observed each time. Data were normally distributed and were presented as mean ± standard deviation (SD). Statistical comparison and *P* values calculation were described early 26, 28.

## Results

### *Gαi3* overexpression in cervical cancer is correlated with poor overall survival

**Figure 1:**
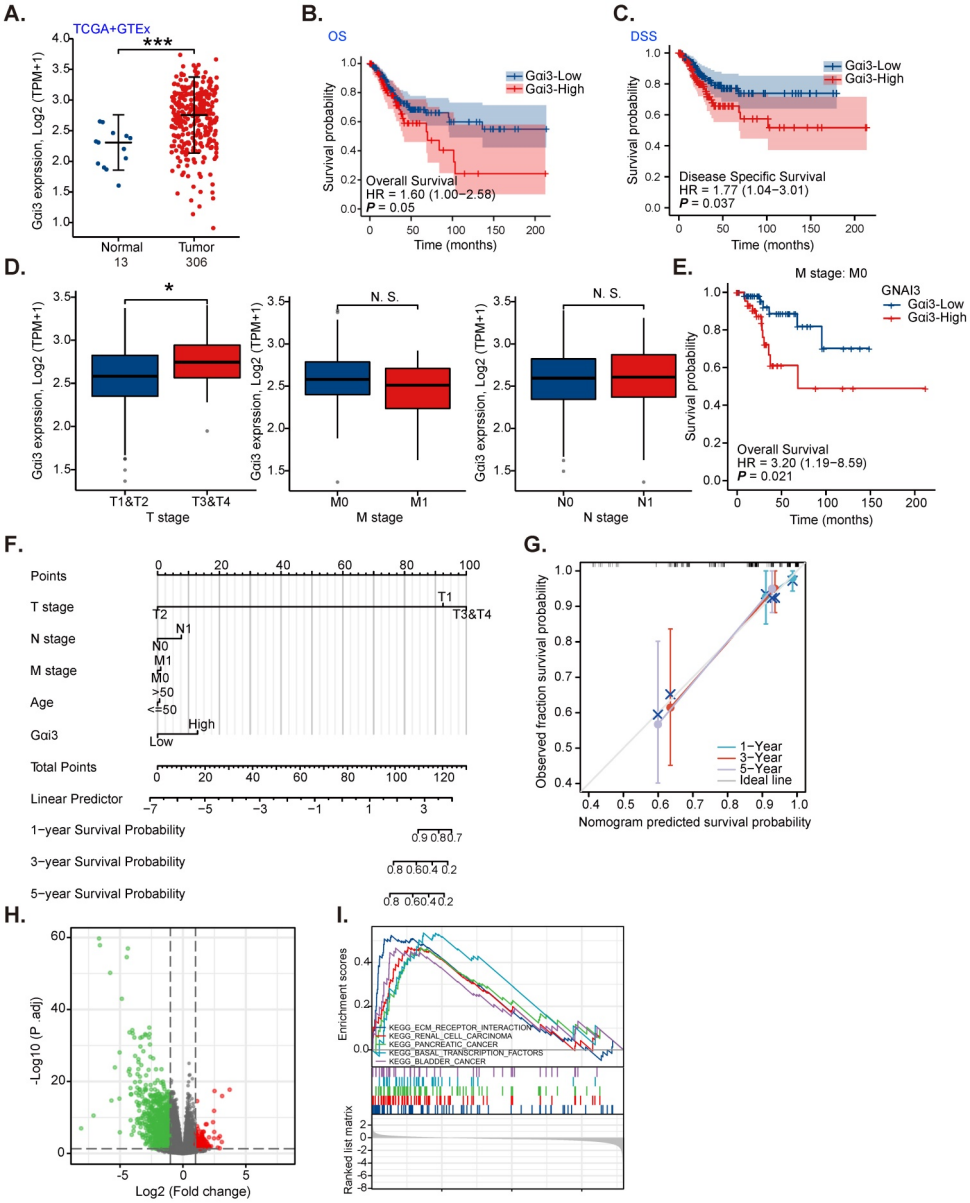
***Gαi3* overexpression in cervical cancer is correlated with poor overall survival.** TCGA cohorts plus GTEx database revealed Gαi3 transcripts in 306 cases of cervical cancer tissues (“Tumor”) and 13 cases of normal epithelial tissues (“Normal”) (**A**). TCGA cervical cancer cohorts (CESC) showed the Kaplan Meier Survival curve of *Gαi3*-low (in blue) and *Gαi3*-high (in red) cervical cancer patients (**B** and **C**). The subgroup analyses of *Gαi3* mRNA expression and clinical characteristics of cervical patients in TCGA cervical cancer cohorts (CESC) were shown (**D** and **E**). Nomogram for high *Gαi3* expression in predicting 1-, 3- and 5-year overall survival probability of cervical cancer patients was shown (**F** and **G**). The volcano map of differentially expressed gene (DEGs) based on *Gαi3* expression in TCGA cervical cancer cohorts (CESC) was shown (**H**); KEGG pathway analysis of *Gαi3*-associated DEGs and enriched pathways were presented (**I**). ***P < 0.001; *P < 0.05; “N. S.” means ***P*** > 0.05.

TCGA and the Genotype-Tissue Expression (GTEx) databases reveal that the number of *Gαi3* (*GNAI3*) mRNA transcripts in cervical cancer tissues (“Tumor”, n= 306) was remarkably higher than it in normal cervical tissues (“Normal”, n = 13) (Figure **1A**). High *Gαi3* expression was correlated with the low overall survival (OS, HR=1.60, ***P*** = 0.05, Figure **1B**) and low disease-specific survival (DSS, HR=1.77, ***P*** =0.037, Figure **1C**). High *Gαi3* expression was significantly correlated with poor prognosis in advanced T-stage cervical cancers (Figure **1D**). *Gαi3* overexpression in cervical cancer was however not associated with M-stage and N-stage status (Figure **1D**). In addition, subgroup analysis of different clinical characteristics showed that *Gαi3* overexpression in M0 cervical cancer patients was significantly associated with poor prognosis (HR=3.20, ***P*** = 0.021, Figure **1E**).

Alignment Diagram (Nomogram) prediction map based on clinical parameters and Gαi3 expression could effectively predict the occurrence probability of 1-, 3-, and 5-year survival response (Figure **1F** and **G**). The high Gαi3 expression predicting poor 1-, 3-, and 5-year survival response is highly consistent with the actual clinical results (Figure **1F** and **G**).

Next, TCGA results were analyzed and the differentially expressed gene (DEGs) were retrieved to examine co-expression genes with *Gαi3* in cervical cancer tissues. The volcanic map of *Gαi3*-assocaited DEGs is shown in Figure **1H** (|LogFC|>1, Adjust ***P***-value < 0.05). KEGG pathway analysis (Figure **1I**) found that *Gαi3*-associated DEGs were enriched in different oncogenic cascades including extracellular matrix (ECM) receptor interaction, renal cell cancer (RCC), pancreatic cancer, basal transcription factors and bladders (Figure **1I**). These results implied that *Gαi3*-associated DEGs could be involved in carcinogenesis and cancer progression. Together, these bioinformatics results show that *Gαi3* overexpression in cervical cancer is correlated with poor overall survival.

### Gαi3 upregulation in cervical cancer tissues of local patients

**Figure 2:**
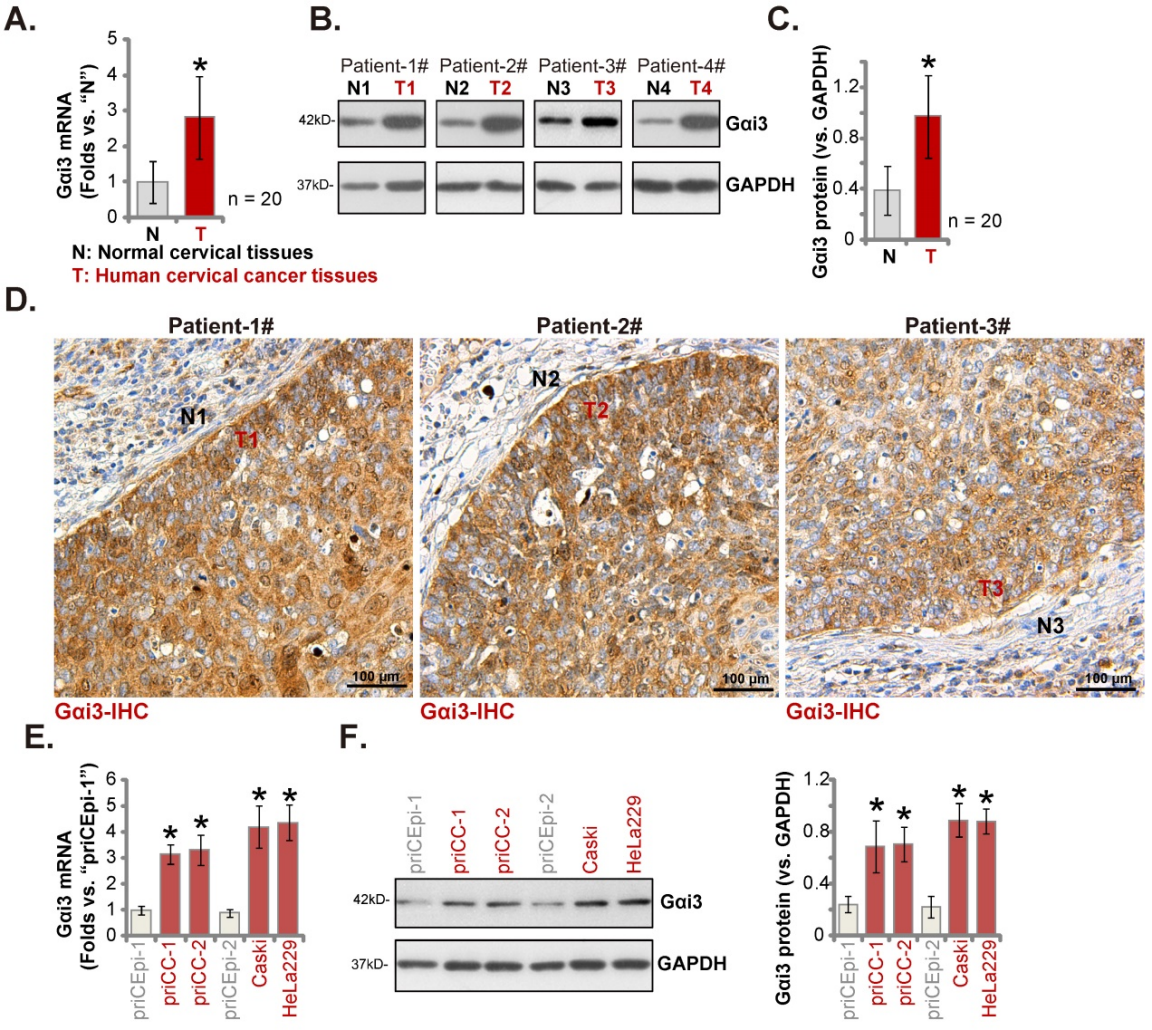
** Gαi3 upregulation in cervical cancer tissues of local patients.** Listed genes and proteins in cervical cancer tissues (“T”) and matched normal cervical epithelial tissues (“N”) from a total of twenty (n = 20) primary cervical cancer patients were measured, and results were quantified (**A-C**). IHC images confirmed Gαi3 protein upregulation in cervical cancer tissue slides of three representative patients (**D**). *Gαi3* mRNA and protein expression in listed cervical cancer cells and cervical epithelial cells was shown (**E** and **F**). *P < 0.05 versus “N” tissues/priCEpi-1 cells. Scale bar = 100 µm.

Next we examined Gαi3 expression in local cervical cancer tissues. The cervical cancer tissues (“T”) and matched paracancerous cervical epithelial tissues (“N”) of twenty (n = 20) primary cervical cancer patients were obtained. *Gαi3* mRNA levels in the cervical cancer tissues were s higher (Figure **2A**). Moreover, Gαi3 protein expression was remarkably elevated in four patients (Patient 1# to 4#)'s cervical cancer tissues (Figure **2B**). When combining all twenty sets patient tissues' blotting data, we discovered that Gαi3 protein upregulation in cervical cancer tissues was significant (Figure **2C**). In addition, the immunohistochemistry (IHC) staining results of Patient 1# to 3# further supported robust Gαi3 protein upregulation in cervical cancer (Figure **2D**). Gαi3 expression in human cervical cancer cells was tested as well. As shown expression levels of both *Gαi3* mRNA and protein were elevated in the primary human cervical cancer cells (“priCC-1” and “priCC-2”) and immortalized lines (Caski and HeLa229) (Figure **2E** and **F**). The relative low Gαi3 expression was obsereved in primary cervical epithelial cells (“priCEpi-1” and “priCEpi-2”) (Figure **2E** and **F**). These results clearly supported Gαi3 overexpression in cervical cancer.

### shRNA-induced silencing of Gαi3 inhibits cervical cancer cell growth and migration

**Figure 3:**
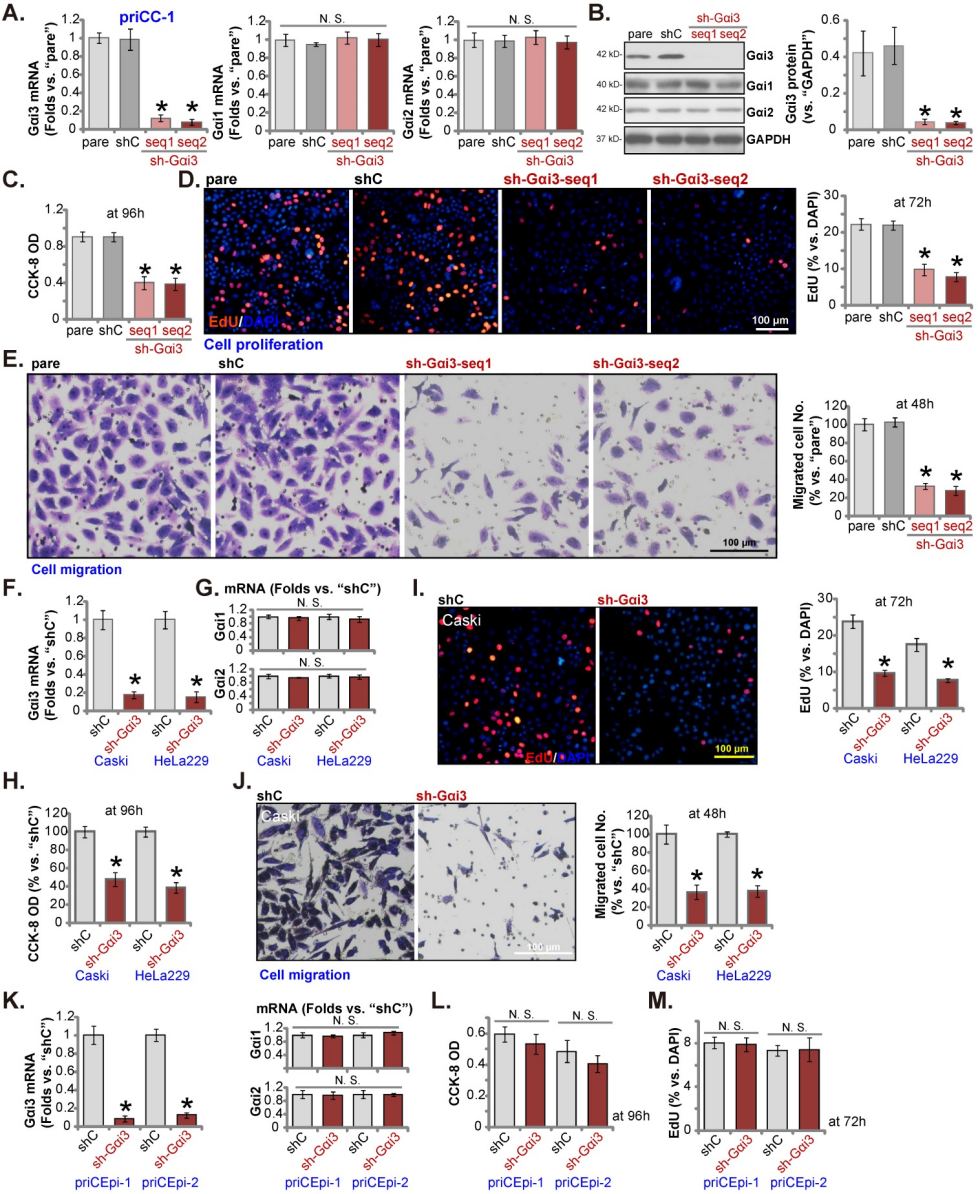
** shRNA-induced silencing of Gαi3 inhibits cervical cancer cell growth and migration.** The primary priCC-1 cells, the established Caski and HeLa229 cells, priCEpi-1 and priCEpi-2 epithelial cells were stably transduced with the applied lentiviral Gαi3 shRNA (“sh-Gαi3”, seq1/seq2 standing for two different sequences) or the scramble control shRNA (“shC”), listed genes and proteins were tested (**A, B, F, G** and **K**). Cells were further cultivated for another 48-96h, cell viability (**C, H,** and **L**), proliferation (**D, I** and **M**), migration (**E** and **J**) were tested. “pare” were the parental control cells (same for all Figures). *P < 0.05 versus “pare”/“shC” group. “N. S.” means ***P*** > 0.05. Scale bar = 100 µm.

In order to test whether Gαi3 could exert pro-cancerous activity, the shRNA strategy was utilized. Two lentiviral Gαi3 shRNAs, “sh-Gαi3-seq1” and “sh-Gαi3-seq2” 26, were transduced to priCC-1 primary cancer cells. Following selection, stable priCC-1 cells bearing Gαi3 shRNA were formed. *Gαi3* mRNA was silenced in sh-Gαi3-bearing stable priCC-1 cells (Figure **3A**). *Gαi1* /*2* mRNA expression was however unchanged (Figure **3A**). Gαi3 shRNAs in priCC-1 cells also resulted in remarkable Gαi3 protein downregulation (Figure **3B**), leaving Gαi1/2 protein expression unaffected (Figure **3B**). CCK-8 OD, or cell viability, was decreased in Gαi3-silenced priCC-1 cells (Figure **3C**). In addition, Gαi3 silencing robustly hindered EdU incorporation and decreased EdU-positive nuclei percentage in priCC-1 cells, causing significant proliferation inhibition (Figure **3D**). Silencing Gαi3 by the targeted shRNAs slowed priCC-1 cell *in vitro* migration (Figure **3E**) assays. These results showed that Gαi3 shRNA provoked significant anti-cancer activity in primary cervical cancer cells.

The established Caski and HeLa229 cells were transduced with sh-Gαi3-seq1, and stable cells formed, namely “sh-Gαi3” cells. The applied Gαi3 shRNA led to significant *Gαi3* mRNA silencing in the immortalized cervical cancer cells (Figure **3G**), and *Gαi1/2* mRNA expression was unchanged (Figure **3H**). Similar to the results in priCC-1 primary cells, Gαi3 silencing inhibited viability (Figure **3H**), EdU incorporation/proliferation (Figure **3I**) and migration (Figure **3J**) in the immortalized cells. The sh-Gαi3-seq1-containing lentivirus was transfected to the primary human cervical epithelial cells, priCEpi-1 and priCEpi-2 (see Figure **2**). The stable cells with the shRNA were established and they were named as “sh-Gαi3” epithelial cells, where *Gαi3* mRNA levels were significantly downregulated (Figure **3K**). *Gαi1/2* mRNA expression was unaffected (Figure **3K**). Interestedly, Gαi3 silencing failed to significantly decrease viability (Figure **3L**) and nuclear EdU incorporation (Figure **3M**) in primary cervical epithelial cells.

### Gαi3 silencing provokes apoptosis in cervical cancer cells

**Figure 4:**
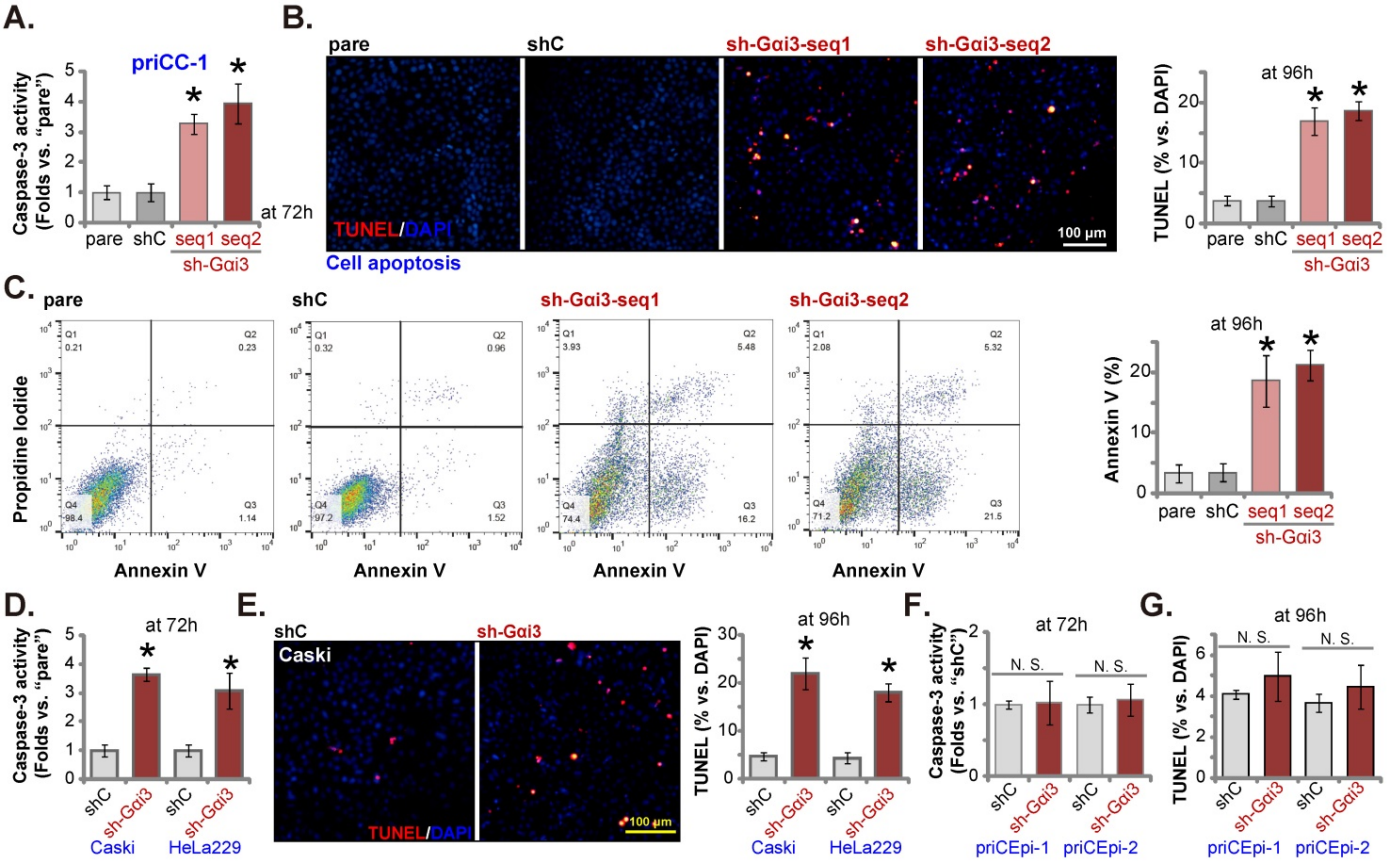
** Gαi3 silencing provokes apoptosis in cervical cancer cells.** priCC-1 cells, the established Caski and HeLa229 cells, priCEpi-1 and priCEpi-2 epithelial cells were stably transduced with the applied lentiviral Gαi3 shRNA (“sh-Gαi3”, seq1/seq2) or the scramble control shRNA (“shC”), cells were further cultured for 72-96h, the Caspase-3 activity was tested (**A, D** and **F**); Cell apoptosis was tested by nuclear TUNEL/DAPI staining (**B, E** and **G**) and Annexin V flow cytometry (**C**) assays. *P < 0.05 versus “shC” group. “N. S.” means ***P*** > 0.05. Scale bar = 100 µm.

Next we examined the potential effect of Gαi3 silencing on cell apoptosis. As shown, in priCC-1 cells expressing Gαi3 shRNA (“sh-Gαi3-seq1” or “sh-Gαi3-seq2”), increased Caspase-3 activity was detected (Figure **4A**). TUNEL-positively stained nuclei (Figure **4B**) and Annexin V-positive cells (Figure **4C**) were significantly boosted after Gαi3 silencing, supporting apoptosis activation. In Caski and HeLa229 cells, shRNA-induced stable knockdown of Gαi3 enhanced the Caspase-3 activity (Figure **4D**) and TUNEL nuclei percentage (Figure **4E**). In priCEpi-1 and priCEpi-2 normal cell silencing of Gαi3, using sh-Gαi3-seq1, failed to augment the Caspase-3 activity (Figure **4F**) and TUNEL-positively stained nuclei number (Figure **4G**).

### Gαi3 KO results in robust anti-cervical cancer cell activity

**Figure 5:**
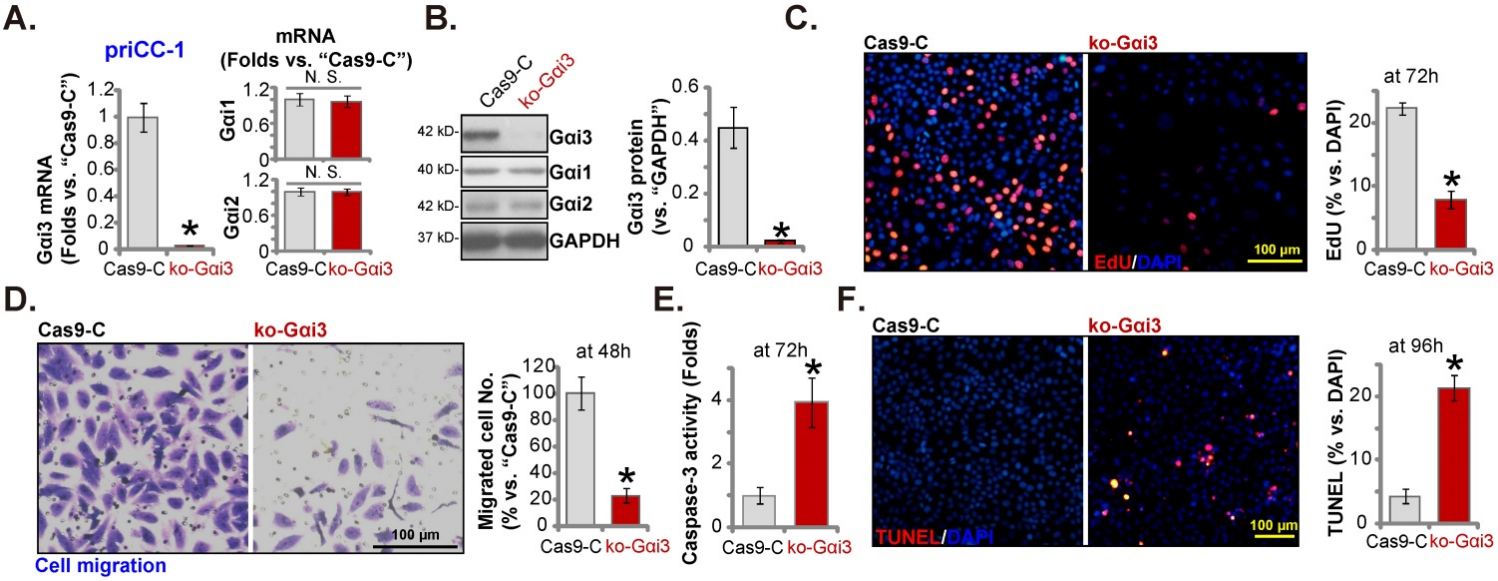
** Gαi3 KO results in robust anti-cervical cancer cell activity.** The primary priCC-1 cells, bearing a lenti-CRISPR/Cas9-Gαi3-KO construct (“ko-Gαi3”) or the empty vector (“Cas9-C”), were established, and listed genes and proteins were measured (**A** and **B**); Cells were further cultured for another 48-96h, cell proliferation and migration (**C** and **D**), as well as the Caspase-3 activity (**E**) and apoptosis (**F**) were tested. *P < 0.05 versus “Cas9-C” group. “N. S.” means ***P*** > 0.05. Scale bar = 100 µm.

To further support the pro-cancerous activity of Gαi3, the CRISPR/Cas9 gene editing strategy, as described 26, was employed to knockout (KO) Gαi3 in cervical cancer cells (“ko-Gαi3” priCC-1 cells). As compared to the control Cas9-expressing priCC-1 cells with the lenti-CRISPR/Cas9 empty vector (“Cas9-C”), Gαi3 was depleted in the ko-Gαi3 priCC-1 cells (Figure **5A** and **B**), where Gαi1/2 expression was unchanged (Figure **5A** and **B**). Gαi3 KO inhibited priCC-1 cell proliferation and reduced EdU nuclei percentage (Figure **5C**). In addition, priCC-1 *in vitro* cell migration (Figure 5**D**) was largely hindered following Gαi3 KO. In the ko-Gαi3 priCC-1 cells, the Caspase-3 activity (Figure 5**F**) and the TUNEL percentage (Figure 5**G**) were both increased. Therefore, Gαi3 KO exerted robust anti-cancer activity in primary cervical cancer cells.

### Gαi3 overexpression exerts pro-cervical cancer activity

**Figure 6:**
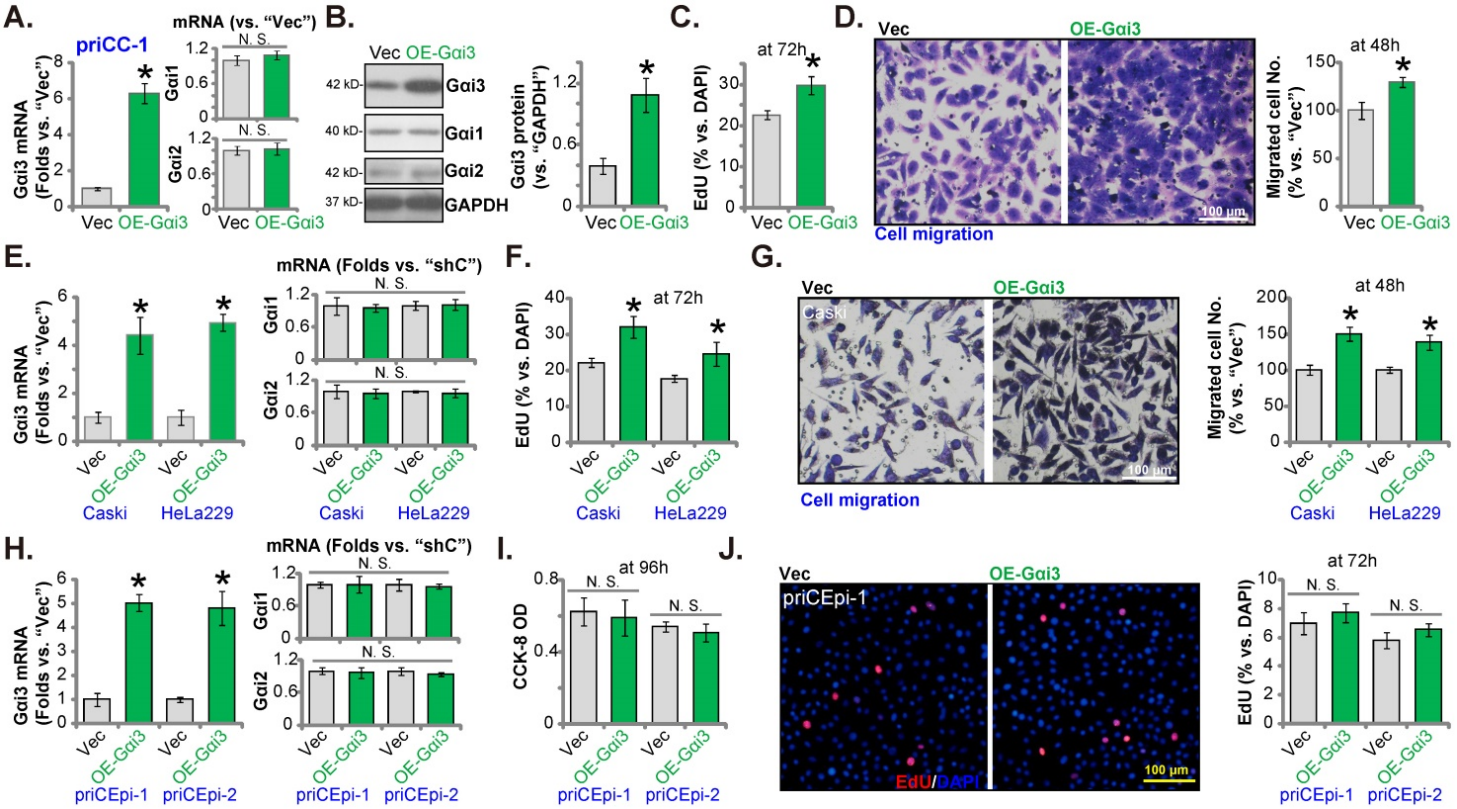
** Gαi3 overexpression exerts pro-cervical cancer activity.** The primary priCC-1 cells, the established Caski and HeLa229 cells, priCEpi-1 and priCEpi-2 epithelial cells, bearing the lentiviral Gαi3-expressing construct (“OE-Gαi3”) or the empty vector (GV369, “Vec”), were established, and expression of listed genes and proteins were tested (**A, B, E** and **H**); Cells were further cultured for 48-96h, EdU incorporation/proliferation (**C, F** and **J**), migration (**D** and **G**), and viability (**I**) were measured. *P < 0.05 versus “Vec” group. “N. S.” means ***P*** > 0.05. Scale bar = 100 µm.

Gαi3 silencing or KO resulted in robust anti-cervical cancer cell activity. Ectopic overexpression Gαi3 could therefore possibly induce opposite activity. A lentiviral Gαi3-expressing construct, as reported in our previous studies 16, 18, 26, was transduced to priCC-1 cells. These cells were named as “OE-Gαi3” cells where Gαi3 expression was robustly increased (Figure **6A** and **B**). Gαi1/2 expression was unchanged (Figure **6A** and **B**). In priCC-1 cells, ectopic Gαi3 overexpression promoted cell proliferation and EdU incorporation (Figure **6C**). Moreover, Gαi3 overexpression accelerated priCC-1 cell *in vitro* migration (Figure **6D**).

The same lentiviral Gαi3-expressing construct (“OE-Gαi3”) were stably transduced to Caski cells and HeLa229 cells, causin *Gαi3* mRNA overexpression (Figure **6E**). *Gαi1/2* mRNA expression was unchanged (Figure **6E**). OE-Gαi3 enhanced EdU incorporation (Figure **6F**) and migration (Figure **6G**) in Caski and HeLa229 cells. The Gαi3-expressing construct was stably transduced to priCEpi-1 and priCEpi-2 epithelial cells. Stable cells, or OE-Gαi3 cells, were formed. *Gαi3* mRNA (but not *Gαi1/2* mRNA) upregulation expression was detected in the OE-Gαi3 epithelial cells (Figure **6H**). However, Gαi3 overexpression exerted no significant effects on CCK-8 viability (Figure **6I**) and EdU incorporation/proliferation (Figure **6J**) in priCEpi-1 and priCEpi-2 cells.

### Gαi3 is important for Akt-mTOR activation in cervical cancer cells

**Figure 7:**
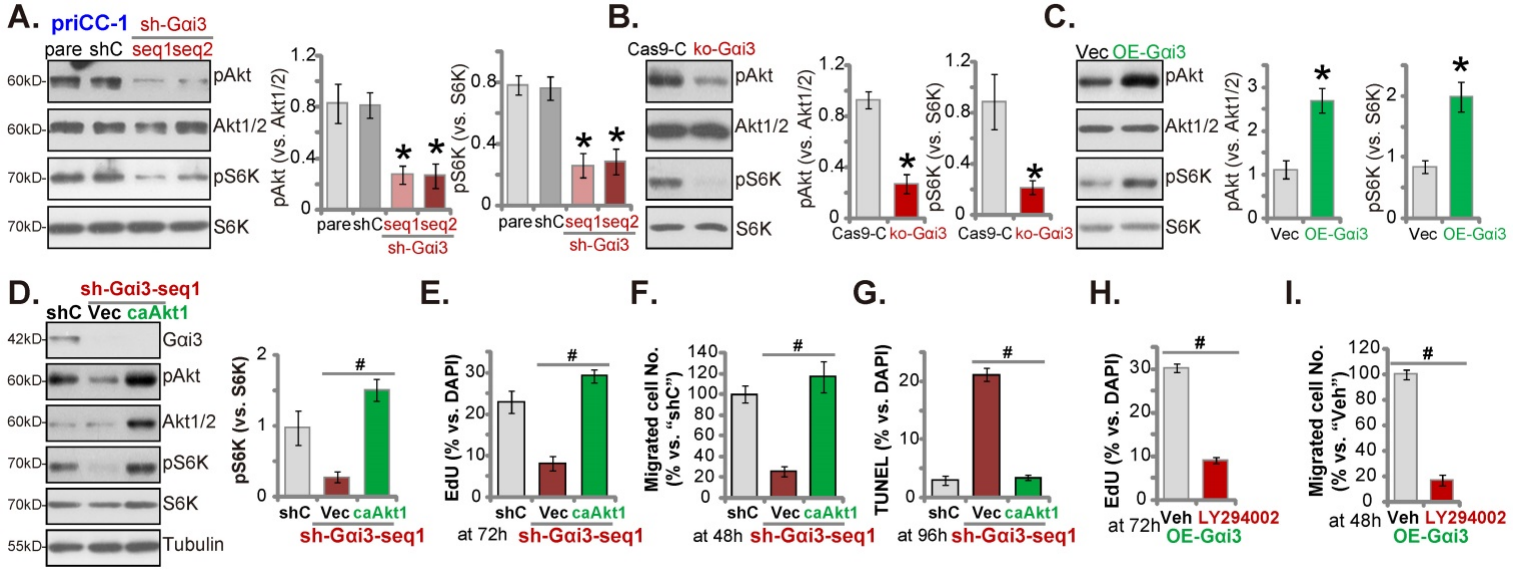
** Gαi3 is important for Akt-mTOR activation in cervical cancer cells.** priCC-1 cells bearing the described genetic modifications were established and culture, and listed proteins in total cell lysates were measured (A-C). Akt-S6K phosphorylation was quantified (**A-C**). The sh-Gαi3-seq1-expressing priCC-1 cells were further stably transduced with a lentiviral constitutively-active Akt1 (caAkt1, S473D) construct or the empty vector (“Vec”), and expression of listed proteins was shown (**D**); Cells were further cultivated for 48-96h, cell proliferation, migration and apoptosis were examined by the described assays, and results were quantified (**E-G**). OE-Gαi3 priCC-1 cells were treated with LY294002 (5 µM) or 0.1% DMSO (“Veh”) for the designated hours, cell proliferation and migration were tested similarly and results were quantified (**H** and **I**). “pare” stands for the parental control cells. *P < 0.05 versus “pare”/“Cas9-C”/“Vec” group. # ***P*** < 0.05. “N. S.” means ***P*** > 0.05.

Gαi proteins association with multiple RTKs (EGFR, VEGFR, TrkB and others 16, 18, 19, 22, 26) is required for mediating downstream Akt-mTOR activation. In priCC-1 cells, shRNA-induced silencing of Gαi3 largely inhibited phosphorylation of Akt (at Ser-473) and S6K (at Thr-389) (Figure **7A**). Moreover, KO of Gαi3 (see Figure **5**) remarkably decreased Akt-S6K phosphorylation in priCC-1 cells. Contrarily, Gαi3 overexpression (OE-Gαi3) in priCC-1 cells enhanced Akt-S6K phosphorylation (Figure **7C**). Akt1/2 and S6K expression levels were unchanged in the Gαi3-altered priCC-1 cells (Figure **7A**-**C**). Gαi3 is therefore important for Akt-mTOR activation in priCC-1 cells.

To test that Gαi3 silencing-provoked anti-cancer cell activity was due to inactivating Akt-mTOR signaling cascade, the lentivirus encoding caAkt1 26) was transduced to sh-Gαi3-seq1-expressing priCC-1 primary cells. caAkt1 completely restored Akt-S6K phosphorylation in the Gαi3-silenced priCC-1 cells (Figure **7D**) without affecting Gαi3 protein expression (Figure **7D**). Importantly, Gαi3 shRNA-induced proliferation inhibition (Figure **7E**), migration reduction (Figure **7F**) and apoptosis (Figure **7G**) were almost completely abolished by caAkt1. Thus Gαi3 silencing-induced anti-cervical cancer cell activity was possibly due to inactivating Akt-mTOR activation. Next, we found that the Akt-mTOR blocker LY294002 32 largely inhibited proliferation (Figure **7H**) and migration (Figure **7I**) of OE-Gαi3 priCC-1 cells.

### GATA4 is important for Gαi3 expression in cervical cancer cells

**Figure 8:**
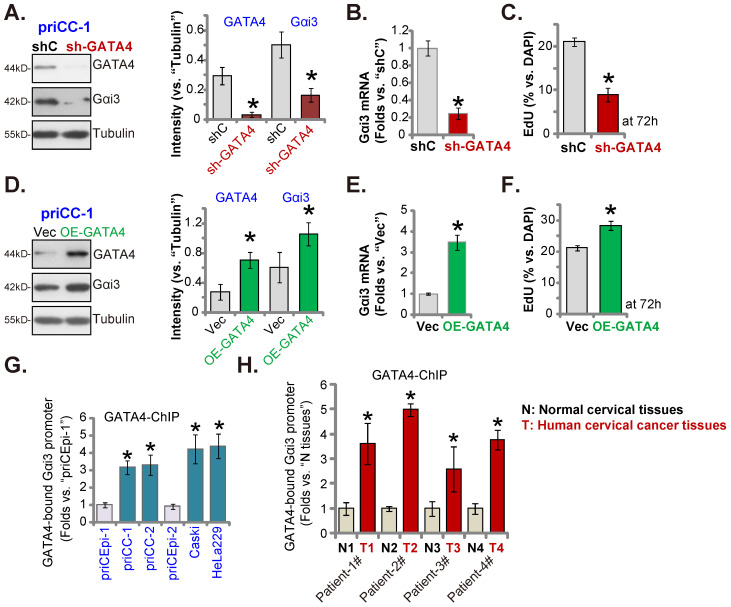
** GATA4 is important for Gαi3 expression in cervical cancer cells.** Expression of listed genes and proteins in primary priCC-1 cells with described GATA4 genetic modification was shown (**A, B, D** and **E**). Cells were further cultivated for 72h and cell proliferation was tested by measuring EdU-positive nuclei percentage (**C** and **F**). Chromosome IP (ChIP) revealed the relative amount of *Gαi3* promoter DNA binding to GATA4 protein in the listed cervical cancer cells and epithelial cells (**G**) as well as in the listed human tissues of four representative patients (**H**). *P < 0.05 versus “shC”/“Vec”/“priCEpi-1 cells”/“N” tissues.

Our group 28 and others have supported that GATA4 is one important transcription factor for *Gαi3* 33. We therefore analyzed whether GATA4 was the primary mechanism of Gαi3 overexpression in cervical cancer. GATA4 shRNA-expressing lentivirus 28 was stably transfected to primary human cervical cancer priCC-1 cells, resulting in robust GATA4 silencing (Figure **8A**). GATA4 shRNA robustly decreased* Gαi3* mRNA and protein (Figure **8A** and **B**) expression in priCC-1 cells. Moreover, GATA4 shRNA inhibited priCC-1 cell proliferation and decreased EdU-incorporated nuclei ratio (Figure **8C**).

On the contrary, the GATA4-overexpressing lentiviral construct was stably transduced to priCC-1 cells to establish OE-GATA4 cells, where GATA4 protein level was remarkably increased (Figure **8D**). OE-GATA4 resulted in *Gαi3* mRNA (Figure **8E**) and protein (Figure **8D**) upregulation in priCC-1 cells. Cell proliferation, tested by EdU incorporation, was enhanced by GATA4 overexpression (Figure **8F**). Therefore, GATA4 is indeed essential for Gαi3 expression in cervical cancer cells.

Remarkably, GATA4 chromosome immunoprecipitation (ChIP) results revealed that GATA4-*Gαi3* promoter DNA binding 33 in cervical cancer cells was robustly higher than that in priCEpi-1 and priCEpi-2 epithelial cells (Figure **8G**). Moreover, in cervical cancer tissues of four representative patients, GATA4 binding to the *Gαi3* promoter DNA was significantly higher than that in the matched surrounding normal cervical tissues (Figure **8H**). These results implied that increased GATA4-*Gαi3* promoter binding could be one primary mechanism of Gαi3 overexpression in cervical cancer.

### Gαi3 depletion suppresses cervical cancer xenograft growth in nude mice

**Figure 9:**
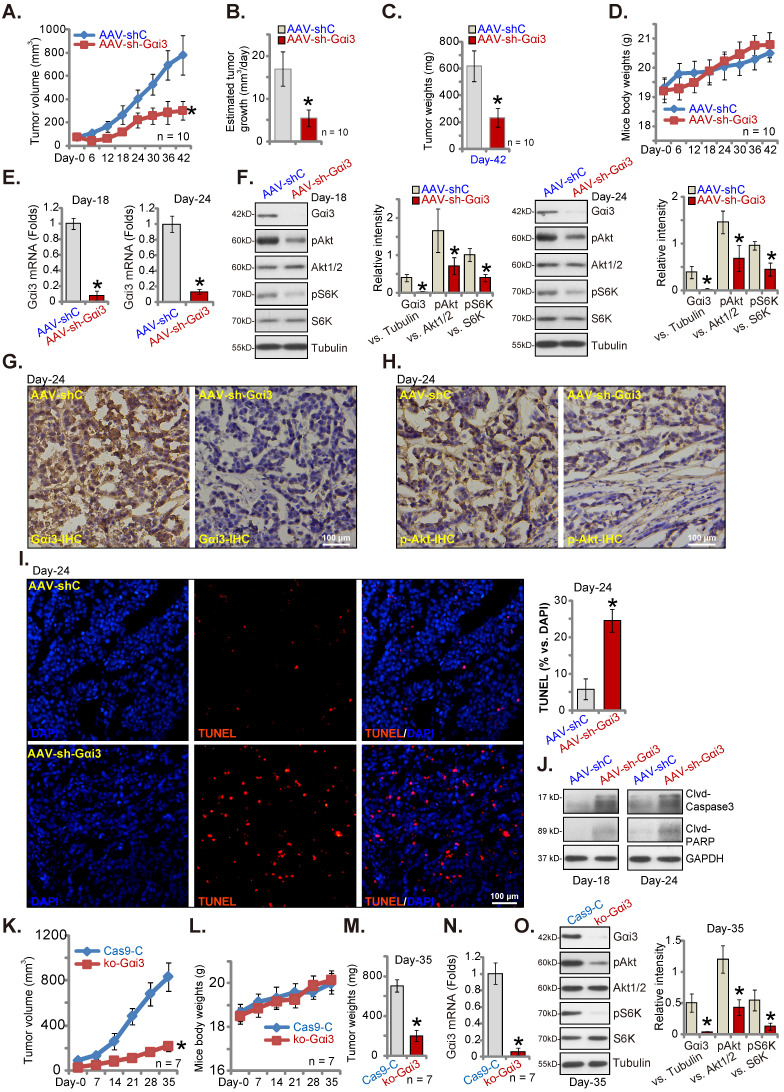
** Gαi3 depletions suppresses cervical cancer xenograft growth in nude mice.** The priCC-1 xenograft-bearing nude mice were intratumorally injected daily with AAV-packed Gαi3 shRNA (AAV-sh-Gαi3) or control AAV shRNA (AAV-shC). Tumor volumes (**A**), the estimated daily tumor growth (**B**), priCC-1 xenograft weights (at Day-42, **C**) and animal body weights (**D**) were shown. At Day-18/Day-24, one priCC-1 xenograft in AAV-sh-Gαi3 and AAV-shC groups was carefully isolated, listed genes and proteins in the xenograft tissue lysates were tested (**E, F** and **J**). The representative IHC images of Gαi3 and p-Akt (Ser-473) were presented (**G** and **H**). The representative fluorescence images showing nuclear TUNEL and DAPI staining in xenograft slides were presented as well (**I**). The ko-Gαi3 priCC-1 cells or the Ca9-C control priCC-1 cells were s.c. injected to nude mice's right flanks. After three weeks, recordings were started (“Day-0”). Tumor volumes (**K**) and the mice body weights (**L**) were presented. At Day-35, priCC-1 xenografts were isolated and weighted (**M**). The listed genes and proteins in the xenograft lysates were tested (**N** and **O**). *P < 0.05 versus “AAV-shC”/“Ca9-C” group. Scale bar = 100 µm.

Gαi3's role on cervical cancer cell growth *in vivo* was explored. priCC-1 cells, at seven million cells per mouse, were* s.c.* injected to nude mice right flanks. After three weeks, the xenografts were formed (~ 80 mm^3^). Mice were then randomly separated into two groups, receiving daily (for ten days) intratumoral injection of AAV-packed Gαi3 shRNA (AAV-sh-Gαi3 26) or control AAV shRNA (AAV-shC 26). Figure **9A** demonstrated that AAV-sh-Gαi3 injection remarkably hindered priCC-1 xenograft growth in nude mice. The estimated daily tumor growth, in mm^3^ per day 26, was remarkably inhibited after AAV-sh-Gαi3 treatment (Figure **9B**). At Day-42, priCC-1 xenografts were isolated and the AAV-sh-Gαi3 group priCC-1 xenografts were lighter than the AAV-shC group xenografts (Figure **9C**). The mice body weights between the two groups were indifferent (Figure **9D**). Thus, intratumoral injection of Gαi3 shRNA-AAV remarkably suppressed priCC-1 xenograft growth.

At Day-18 and 24, one priCC-1 xenograft in the treatment and control group mice was carefully isolated (total four xenografts). Signaling proteins were tested. Western blotting and qRT-PCR assaying of fresh tissue lysates found that *Gαi3* mRNA (Figure **9E**) and protein (Figure **9F**) were silenced in AAV-sh-Gαi3-injected xenografts. Moreover, Akt-S6K phosphorylation was significantly decreased (Figure **9F**). The representative IHC images further confirmed Gαi3 protein silencing in AAV-sh-Gαi3-injected priCC-1 xenografts (at Day-24, Figure **9G**). In addition, IHC images verified Akt inhibition in Gαi3-silenced priCC-1 xenografts (at Day-24, Figure **9H**). The fluorescence staining of priCC-1 xenograft slides demonstrated increased TUNEL staining in AAV-sh-Gαi3-injected tumors (at Day-24, Figure **9I**), indicating apoptosis activation. Moreover, Caspase-3 and PARP cleavages were increased (Figure **9J**). Thus, Gαi3 silencing by AAV-sh-Gαi3 injection robustly suppressed Akt-mTOR activation and provoked apoptosis in priCC-1 xenografts.

To further support the important role of Gαi3 in cervical cancer cell growth *in vivo*, ko-Gαi3 priCC-1 cells or “Cas9-C” cells (see Figure **5**) were *s.c.* injected to nude mice's flanks. The tumor recordings were started three weeks after (labeled as “Day-0”). The growth of ko-Gαi3 priCC-1 xenografts was largely inhibited when compared to the Cas9-C priCC-1 xenografts (Figure **9K**). Animal body weights were again indifferent (Figure **9L**). At Day-35 we found that Gαi3 KO priCC-1 xenografts were much lighter than Cas9-C xenografts (Figure **9M**).* Gαi3* mRNA and protein were depleted in ko-Gαi3 priCC-1 xenografts (Figure **9N** and **O**), where Akt-S6K phosphorylation was remarkably decreased (Figure **9O**).

## Discussion

The mortality rate of cervical cancer has obvious regional differences 3, 4, 34. Traditional treatment methods, including surgery, radiotherapy and chemotherapy, have their limitations. With the latest development of targeted therapy, especially the use of targeted drugs such as bevacizumab, the survival time of certain cervical cancer patients could be prolonged 7, 9, 12. However for most cervical cancer patients, the novel targeted therapies are in urgent need 7, 9, 12.

Our recent studies have shown that Gαi3 could be a novel therapeutic oncotarget of human cancer 16, 26. We previously found that Gαi3 expression is elevated in osteosarcoma and correlates with poor overall survival 26. Gαi3 is important for osteosarcoma cell growth Conversely, Gαi3 shRNA or KO robustly inhibited osteosarcoma cell growth 26. Moreover, Gαi3 overexpression was detected in human glioma tissues and is significantly correlated with poor overall survival 16. Gαi3 silencing potently inhibited patient-derived glioma xenograft orthotopic growth 16. Overexpression of Gαi3, on the other hand, significantly enhanced glioma growth 16.

Our study supports that Gαi3 is a valuable oncotarget of cervical cancer. The bioinformatics analysis revealed that the number of *Gαi3* mRNA transcripts is elevated in human cervical cancer tissues, and *Gαi3* upregulation was correlated with patients' poor overall survival and DSS. *Gαi3* mRNA and protein levels in local cervical cancer tissues were upregulated. Remarkably, Gαi3 depletion resulted in robust anti-cervical cancer cell activity. In different cervical cancer cells, Gαi3 silencing or KO resulted in robust anti-cancer activity. Conversely, ectopic overexpression of Gαi3 further promoted cervical cancer proliferation. *In vivo*, the growth of cervical cancer xenografts was remarkably hindered after Gαi3 silencing or KO. These results clearly supported that targeting Gαi3 could be a promising therapeutic strategy against cervical cancer.

Our group has identified Gαi proteins, Gαi1 and Gαi3, as key signaling molecules mediating downstream signalings by a number RTKs 16, 18-22, 25, 26, 28 and non-RTK receptors 17. Gαi1 and Gαi3 can associate with ligand-activated RTKs to transduce downstream mitogenic/oncogenic signaling cascades 16, 18-22, 25, 26, 28. For example, Gαi1/3 located in the VEGFR2 endocytosis complex, essential for VEGF (vascular endothelial growth factor)-induced endocytosis of VEGFR2 and downstream signaling transduction 18. Moreover, Gαi1/3 proteins are required for brain-derived neurotrophic factor (BDNF)-induced signaling activation 19. In addition, Gαi1/3 proteins can associate with EGF-stimulated EGFR and the adaptor protein Gab1, mediating downstream Akt-mTOR cascade activation 22.

The Akt-mTOR signaling is an important target for the development of cervical cancer therapeutics 35, 36. Here we found that Gαi3 is vital for Akt-mTOR cascade activation in cervical cancer cells. Akt-mTOR activation was significantly inhibited after Gαi3 silencing or KO, but augmented following Gαi3 overexpression. Reduced Akt-S6K phosphorylation was also detected in cervical cancer xenograft tissues with Gαi3 silencing or KO. Notably, Gαi3 silencing-induced anti-cervical cancer activity, including proliferation inhibition, migration reduction and apoptosis induction, were almost reversed following Akt-S6K re-activation by caAkt1. Moreover, Gαi3 overexpression-induced proliferation and migration acceleration was largely inhibited by LY294002, the PI3K-Akt-mTOR inhibitor, in cervical cancer cells.

An early study using luciferase reporter assay and chromatin Immunoprecipitation (ChIP) demonstrated that the transcription factor GATA4 can directly bind to the promoter region of *Gαi3* and regulate its transcriptional activity and expression 33. The very recent study of our group has shown that GATA4 is an important transcription factor of *Gαi3* in endothelial cells 28. We further discovered that phosphoenolpyruvate carboxykinase 1 (PCK1) associated with phosphorylated GATA4, promoting Gαi3 transcription and expression in endothelial cells 28. It will then lead to increased Akt-mTOR activation and pro-angiogenesis response 28. Here in cervical cancer cells, Gαi3 was decreased following GATA4 shRNA, but was upregulated following GATA4 overexpression. Significantly, an increased binding between GATA4 and *Gαi3* promoter region in both cervical cancer tissues and various cervical cancer cells was detected. These results implied that GATA4-mediated increased *Gαi3* transcription could be a primary mechanism of *Gαi3* upregulation in cervical cancer.

## Conclusion

These data suggest that targeting Gαi3 would be a promising therapeutic strategy against cervical cancer. Specific pharmacological inhibitors or pipeline drugs blocking Gαi3 association with RTKs should then inhibit downstream oncogenic cascade activation and cervical cancer progression.

## Supplementary Material

Supplementary figures.

## Figures and Tables

**Table 1:** The information of the cervical cancer patients

No.	Age	Stage	Differentiation	Left lymph node	Right lymph node	Metastasis	P16	Ki67
1	29	IB2	High	0/6	0/8	no	+	+, 67%
2	42	IIIC1p	Mid to Low	0/7	1/9	no	+	+, 85%
3	52	IB1	High	0/10	0/10	no	+	+, 50%
4	69	IB1	High	0/9	0/10	no	+	+, 75%
5	40	IIA1	Mid	0/6	0/10	no	+	+, 90%
6	49	IB2	Mid to Low	0/12	0/11	no	-	+, 70%
7	62	IA1	Low	NA	NA	no	NA	NA
8	40	IIA1	Mid	0/6	0/10	no	+	+, 90%
9	35	IB1	High	O/11	2/19	no	+	+, 20%
10	57	IIA2	Mid	0/8	0/17	no	+	+, 70%
11	48	IB2	Low	NA	NA	no	NA	NA
12	44	IIA2	Mid	0/11	0/10	no	NA	NA
13	34	IIA	Low	2/5	0/14	no	NA	NA
14	42	IIA1	Low	0/11	0/16	no	NA	NA
15	64	IIA	Low	0/6	0/6	no	NA	NA
16	48	IIA2	Low	2/5	2/7	no	NA	NA
17	57	IA1	High	NA	NA	no	NA	NA
18	55	IIA1	Low	0/10	0/16	no	NA	NA
19	44	IA2	Low	NA	NA	no	NA	NA
20	27	IB2	High	0/8	0/14	no	NA	NA

“NA” stands for not available.
