# Supplementary material for: Identification of Gαi3 as a novel molecular therapeutic target of cervical cancer
Source: Int J Biol Sci. 2022 Sep 6;18(15):5667–80. doi: 10.7150/ijbs.77126 (PMC9576524; doi:10.7150/ijbs.77126)

Figure S1: The uncropped blotting images of the study.

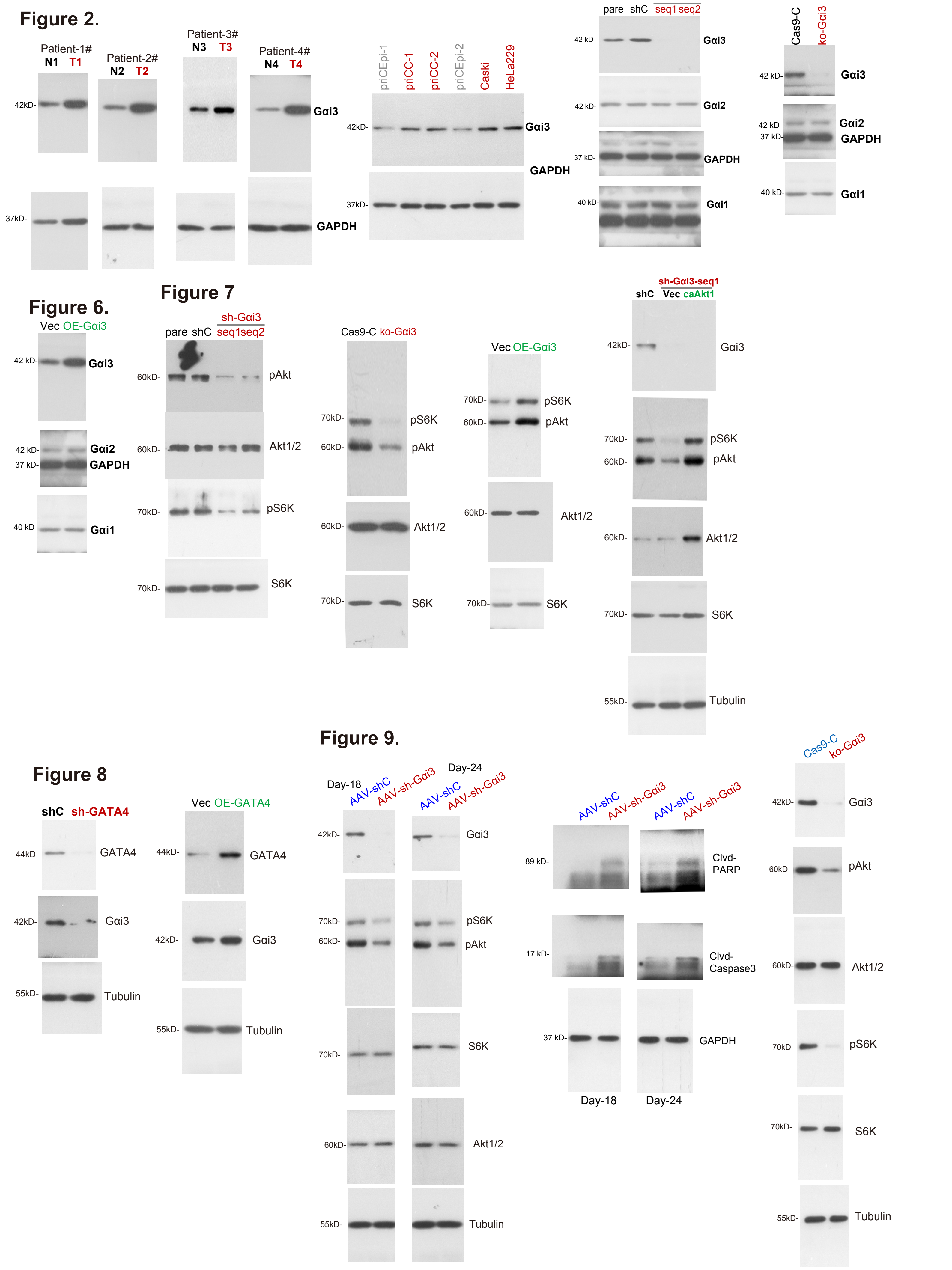

Supplement: Supplementary file 1 — Supplementary figures. [file ijbsv18p5667s1.pdf]
